# Radiopharmaceutical extravasations: a twenty year mini-review

**DOI:** 10.3389/fnume.2023.1219202

**Published:** 2023-07-20

**Authors:** Dustin R. Osborne

**Affiliations:** Department of Radiology, University of Tennessee Graduate School of Medicine, Knoxville, TN, United States

**Keywords:** molecular imaging, extravasation, radiopharmaceuticals, radiopharmaceutical therapy, theranostics

## Abstract

Interest and research into radiopharmaceutical extravasation concepts has risen with the increase in use of radiopharmaceutical therapies, growing access to novel molecular imaging agents, and recent regulatory controversies. This mini-review will examine the literature of the last twenty years to summarize the history of radiopharmaceutical extravasations, determine key trends in imaging and therapies, and highlight critical gaps in research that currently exist. The intent of this work is to provide a summary of this complex topic that helps build awareness and promotes new innovations in this interesting aspect of theranostic radiopharmaceuticals.

## 1. Introduction

### 1.1. Mini-review objective

Mini-reviews typically begin with a summary of what topic they will cover and their primary topic, however, given the recent controversies around the topic of radiopharmaceutical extravasation, this review will begin with what it is not.

• This mini-review is **not** meant to take a position regarding what type of extravasation monitoring should be performed.
• This mini-review is **not** meant to provide support for, or oppose, the Nuclear Regulatory Commission’s decision to review radiopharmaceutical extravasations (1).

What this mini-review will provide is a brief history of radiopharmaceutical extravasation, review the current landscape of radiopharmaceutical extravasation literature, and highlight key gaps in current research related to this topic.

### 1.2. Summary of radiopharmaceutical extravasations

Radiopharmaceutical extravasations are a potential complication in molecular imaging studies that occurs when a radiopharmaceutical leaks out of the injection site into the surrounding tissue. They occur due to a wide range of reasons from the use of sub-optimal injection techniques to patients simply having poor or degraded vein quality resulting in post-injection veinous rupture (2). These events have historically been considered to be of little concern in molecular imaging, however, the emergence and widespread adoption of new radionuclide therapies have brought increased awareness regarding injection impact for both diagnostic and therapeutic radiopharmaceuticals.

Early awareness of extravasation risks associated with non-radioactive contrast media was shown in studies even back to the 1960s (3), however, the advent of Computed Tomography (CT), Magnetic Resonance Imaging (MRI), and the use of infused chemotherapy drugs for the treatment of cancer brought the analysis of extravasations and their potential clinical impact to the forefront between 1970s and 1980s (4–6). Extravasation remains a key area of interest within MRI and CT appearing consistently in continuing education articles for healthcare providers while radiopharmaceutical extravasations have only gained significant attention more recently. Although an increase in the volume of radionuclide extravasation has been observed, a search of PubMed over the last twenty years for the specific terms “iodinated contrast media extravasation” and “radiopharmaceutical extravasation” shows a ratio of 146 to 11 peer reviewed publications, respectively.

The Nuclear Regulatory Commission (NRC) requires licensees to report all medical events involving the administration of radiopharmaceuticals that result in a radiation dose to the patient exceeding certain levels. However, according to NRC Federal Register documentation, extravasations and infiltrations of radiopharmaceuticals are not classified as medical events and are thus exempt from reporting requirements unless they meet specific criteria (1). Despite the exemption to federal reporting and disagreements on exact processes related to extravasations, many healthcare professionals and regulatory bodies still recommend reporting and documenting radiopharmaceutical extravasations as part of good clinical practice (7–10). Guidances, such as the IAEA Quanum 3.0 even provides specific regulatory guidance for standard operating procedures that should be in place for appropriate management of events that may occur (11). It should be noted that while these guidelines and recommendations suggest reporting extravasations as part of good clinical practice, the specific reporting requirements may vary depending on the regulatory bodies and institutions overseeing the clinical practice setting.

Over the last five years, radiopharmaceutical extravasations have become an unexpected source of controversy within the molecular imaging community. It is well known that non-radioactive pharmaceuticals and contrast agents that are extravasated have potential for significant deleterious effects to patients (12–14). Within the field of nuclear medicine and molecular imaging, however, the working assumptions have generally followed those of the 1980–2002 Federal Register Notices by the NRC that excluded radiopharmaceutical extravasations from reporting requirements as medical events. The NRC in conjunction with the Advisory Committee on the Medical Use of Isotopes (ACMUI) has cited key reasons to exclude radiopharmaceutical extravasations which have been fundamentally true in the past, including: difficulty measuring and quantifying extravasation metrics, limited anticipated safety concerns from radiopharmaceuticals, and the perception that extravasations were essentially unavoidable.

Peer-reviewed studies have highlighted the prevalence of radiopharmaceutical extravasations and the importance of appropriate injection techniques to reduce their incidence (15–24). In addition, dosimetry studies have shown that the impact of radiopharmaceutical extravasations on radiation exposure to patients has potential to be significant, particularly in the case of therapeutic radiopharmaceuticals (25–30). While reporting requirements for radiopharmaceutical extravasations may be controversial at this time, the importance of prompt recognition and appropriate management of these events cannot be overstated. Improved injection techniques, prompt recognition through increased awareness, and improving documentation of extravasations can help minimize the potential risk of complications and radiation exposure to patients.

## 2. Mini-review literature summary

**Figure 1:**
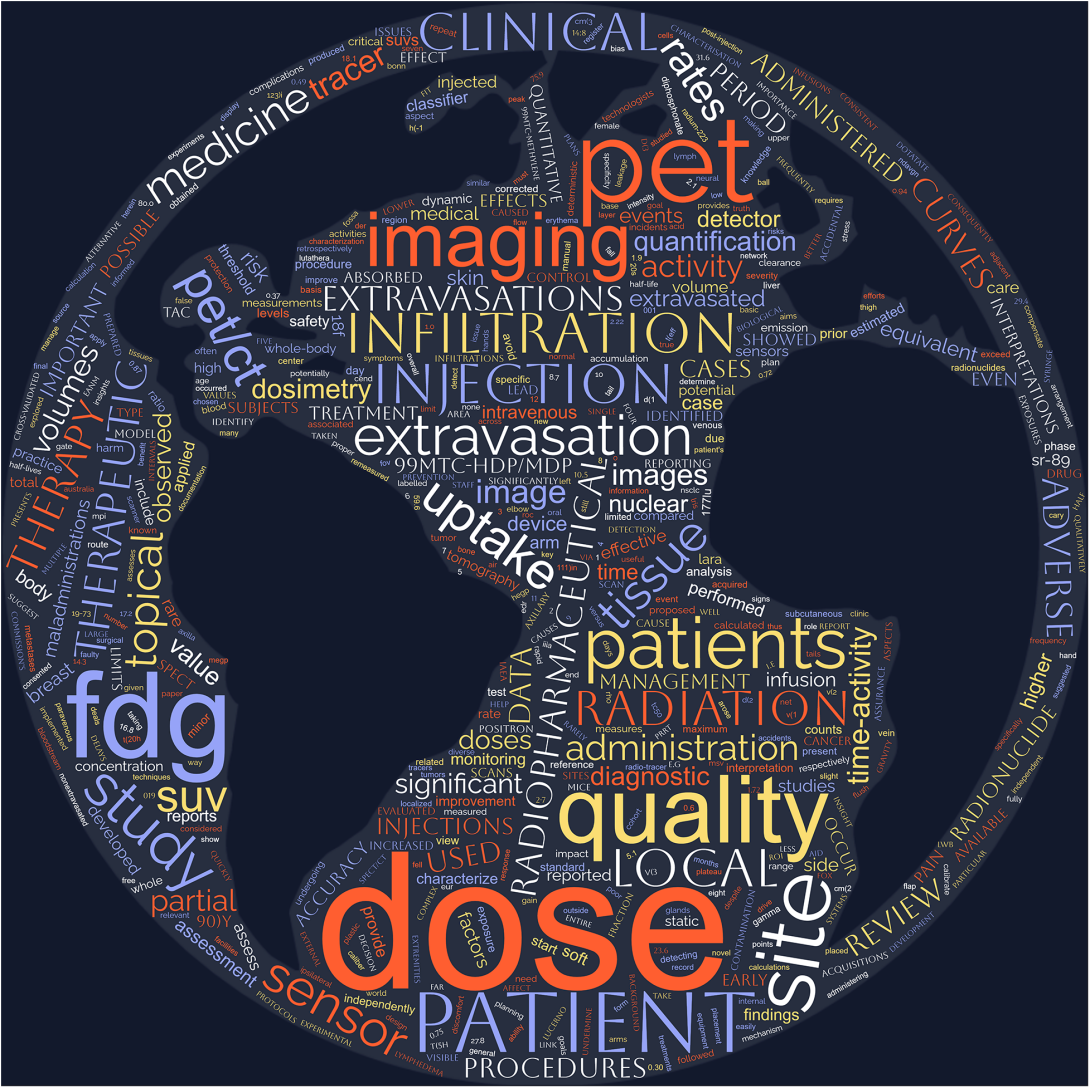
Word cloud image representing frequency of word use in abstracts of publications on radiopharmaceutical extravasation over the last twenty years.

For this mini-review, PubMed was searched using the keywords and search terms highlighted in Table 1 with publication dates from the last twenty years (between 2003 and 2023). Initial search results yielded 512 publications, of which sixty-eight remained after removing duplicates and those articles not pertaining to radiopharmaceutical extravasations. Out of the sixty-eight publications, only two in the last twenty years were qualitative literature reviews assessing reporting prevalence of extravasation events in peer-reviewed publications. Most recently, van der Pol et al. published an extensive review of radiopharmaceutical extravasation literature and this review will not repeat that effort but will instead focus on highlighting recent trends and key gaps in research related to radiopharmaceutical extravasations (23). Figure 1 shows a word cloud rendering created from analysis of the frequency of words used in available reviewed publication abstracts providing a visual representation of common themes.

**Table 1:** PubMed keyword search (2003–2023).

Search string	No. of results
Radiopharmaceutical extravasation	136
Radionuclide + extravasation	156
Nuclear medicine + extravasation	403
PET + extravasation	100
Positron emission tomography + extravasation	93
FDG + extravasation	41
SPECT + extravasation	44
Tc99m + extravasation	10
MDP + extravasation	6
HDP + extravasation	2
F18 + extravasation	14
Dose infiltration	18
Radiopharmaceutical misadministration	9

Only eight (12%) of the studies reviewed sample sizes of 100 patients or more and many of the publications were simple case studies of single events being reported (n=24, 35%). Diagnostic studies made up the majority of reports (n=36, 53%) followed by radionuclide therapy observations (n=17, 25%) and finally studies including both occurrences (n=9, 13%). Across the peer-reviewed research studies, a total of 9,786 patients were included with a total of 784 patients with an extravasation event recorded. The calculated average rate was eight percent across this group of publications and matches well with previous literature reviews and review articles that reviewed historic publications outside the scope of this work. This shows good consistency across independent assessments for a standardized rate of extravasation within these patient populations.

The depth of information related to extravasation occurrence, prevalence, and impact remains severely limited, however, there is a clear trend in increasing numbers of publications. In the first ten years reviewed there were a total of sixteen publications on extravasations and fifty-two in the subsequent ten year period representing growth of 225%. This potentially correlates with increased awareness and interest in this topic. Of particular note is an increase in the number of literature reviews and analyses of larger patient populations which help to enable more global assessment of common extravasation issues (21,23). Increased reporting through publications from institutions is critical to understanding the true impact of extravasations for both diagnostic and therapeutic radiopharmaceuticals.

There is also a notable trend in the creation and validation of tools designed to assess dosimetry related to extravasation events. Six of the sixty-eight publications included methodologies or tools for assessment of radiation dose to the patient at the site of the event representing approximately 9% of publications. Prior to 2017, only one publication had assessed extravasation dosimetry with four new publications on this topic since 2020. This is a growing area of interest both for the purpose of confirming diagnostic injection safety but to also have defined methods and tools for assessment of therapeutic events when they occur (25,26,28,30,31).

Common themes were observed across the studies reviewed in this work, but these commonalities also lead to additional unresolved questions.

The three most common elements were:

• Extravasations are a common clinical occurrence: The average rate of extravasation observed in this review was approximately eight percent. Using the European Medical Association definition for adverse effects, an occurrence of an event 1%–10% would be considered “common” (32,33). Assuming recent estimates for diagnostic nuclear imaging procedures is correct (13.5 million), then that indicates approximately one million patients per year may experience an extravasation event (34). This begs the question, “why is there a significant difference between reported infiltration rates for radiopharmaceutical injections (∼1%–8%) and chemotherapies (up to 6.5%), and why are reported rates far higher than those reported for modalities like CT and MRI (0.045%, 0.26%)? (13,16,18–20,24,35)”
• Extravasations can have clinical impact: Both peer-reviewed and case study publications showed myriad examples of altered uptake and changes to quantitative results. Although most studies noted the importance of documenting a clinical occurrence of extravasation, there is no specific guidance related to when repeat imaging is necessary or at what level of extravasation negatively impacts diagnostic results or therapies. These are critical questions to prevent unnecessary repeat imaging or re-dosing of an individual.
• Diagnostic radiopharmaceuticals do not appear to be of significant concern for radiation exposure: The majority of publications involving diagnostic radiopharmaceuticals did not examine dosimetry or long-term follow-up of patients post extravasation, however, one recent paper performed a retrospective review of extravasation events related to 99mTc-MDP reported via their standard clinical reporting mechanisms showing no significant trends of adverse events related to diagnostic nuclear medicine studies (21). Publications reviewed tend to note that diagnostic radiopharmaceuticals are not expected to result in significant radiation exposure to the patient. Three publications have reported radiation extravasation injury from 201Tl-thallous chloride and 131I-iodocholesterol injections, but both are now sparsely used radiopharmaceuticals and those publication dates were outside the dates examined as part of this mini-review (36–38). A small number of publications have recently examined absorbed doses from diagnostic radiopharmaceutical extravasations or created tools for such calculations which may help in improving standardization of methodology and increased reporting of dosimetry in future case studies (25,26,28,30,31).

## 3. Key gaps in current research

While an increase in publications over the last twenty years suggests an increase in awareness, many fundamental questions related to radiopharmaceutical extravasation events remain unanswered.

1. **Standardization of reporting:** Standardized reporting methods for radiopharmaceutical extravasations do not exist, which makes it difficult to compare data across studies and accurately assess the prevalence and impact of these events. Guidance documents that mention extravasations often only indicate that the event should be noted or was present, however, even the most rigorous quantitative recommendations only additionally suggest attempting to quantify residual activity at the site (7,9). Little guidance is found in the literature regarding the most appropriate or recommended consistent methodology for determination of the residual activity at the injection site in the event of extravasation.
2. **Impact on patient outcomes:** While studies have reported on the occurrence and prevalence of radiopharmaceutical extravasations, there is a lack of data on the true impact of these events on patient outcomes, such as changes in diagnosis or treatment plans in true clinical trials. Although it is generally accepted that extravasations of therapeutic radiopharmaceuticals can be of significant concern, little consideration is given to the various impacts that the extravasation of a diagnostic agent can have on changes to biodistribution or image quantification. This specific theme will require a greater number of independent publications with larger populations to truly determine the overall impact on image quality and quantification.
3. **Extravasation management:** Limited validated information exists on the best management strategies for radiopharmaceutical extravasations and interested practitioners will find conflicting viewpoints on basic tenants such as the use of a warm or cold compress. A warm compress is typically used to disperse the drug to a larger area while cold compresses are used to reduce flow and limit the spread of the drug to the site of injection(39). Radiopharmaceuticals use a wide range of molecules and it is possible that the appropriate strategy may vary with the radiolabeled drug used during a study. No publications were found examining these possible differences nor are there any publications related to radiopharmaceutical extravasations that validate one extravasation resolution over another.
4. **Local Extravasation Dosimetry:** Only seven studies out of the sixty-eight publications reviewed were related to calculation of dosimetry related to extravasations. Although it historically has been widely believed that diagnostic radiopharmaceutical extravasations would impart little dose to the injection site tissues, limited information is available from peer reviewed publications or clinical studies. No publications seem to exist that examine these effects in a prospective manner. Recently, new tools and assessment methodologies have been published or validated to aid in calculation of expected extravasation dosimetry and more work is needed to fully validate extravasation radiation exposure concerns for diagnostic and therapeutic radiopharmaceuticals (25,26,27,28,29,30,31). Although these recent works show absorbed doses can be higher than expected, there is little information on how those absorbed doses translate to equivalent doses at the injection site, which is the most important element for patient safety concerns.
5. **Thresholds for determination of critical events:** Thresholds for critical events will vary depending on whether the radiopharmaceutical is diagnostic or therapeutic. Very little guidance can be found in literature related to parameters for when repeat imaging is necessary due to a compromised scan. In practice, this generally is at the discretion of the radiologist to determine whether the image quality is sufficient or compromised in such a way as to warrant repeat imaging (40). Several studies have looked at extravasation correction techniques or noted how extravasations impact the final reconstructed SUVs, however, none of these methods are currently used widely in the clinical setting and have not been fully vetted in robust trials (41,42). For radiotherapeutics, where imaging may not be standard practice, the assessment of whether to re-treat is based solely on post-therapy imaging where standard lesion progression methods are typically used. Although guidance from international organizations recommends dosimetry calculation for each patient, which would quickly show significant extravasation issues during post-therapy imaging, the practice is so limited in clinical use that guidance documents provide lookup tables for dose estimates that can be used for estimates without imaging (43). More robust guidelines and criteria for when to repeat molecular imaging studies or therapeutic injections are needed.

## 4. Conclusions

A review of literature from the last twenty years reveals an increase in publications related to radiopharmaceutical extravasations. This suggests growing awareness of these events and their potential impact on patient care for imaging and therapy studies. Even with this increased volume of publications, there is still severely limited information related to principles that practitioners can apply in the clinic to make diagnostic and therapeutic decisions for their patients when these events occur. More research is needed across a wider range of radiopharmaceuticals and patient populations worldwide to determine consensus practice suggestions and to determine true clinical impact on imaging studies that use radiopharmaceuticals.
